# Multi-dimensional TOF-SIMS analysis for effective profiling of disease-related ions from the tissue surface

**DOI:** 10.1038/srep11077

**Published:** 2015-06-05

**Authors:** Ji-Won Park, Hyobin Jeong, Byeongsoo Kang, Su Jin Kim, Sang Yoon Park, Sokbom Kang, Hark Kyun Kim, Joon Sig Choi, Daehee Hwang, Tae Geol Lee

**Affiliations:** 1 Center for Nano-Bio Measurement, Korea Research Institute of Standards and Science, Daejeon, Republic of Korea; 2 Department of Nano and Bio Surface Science, University of Science and Technology, Daejeon, Republic of Korea; 3 School of interdisciplinary bioscience and bioengineering, POSTECH, Pohang, Republic of Korea; 4 Department of New Biology, DGIST, Daegu, Republic of Korea; 5 Department of Biochemistry, Chungnam National University, Daejeon, Republic of Korea; 6 National Cancer Center, Goyang, Republic of Korea; 7 Center for Systems Biology of Plant Senescence and Life History, Institute for Basic Science, Daegu, Republic of Korea

## Abstract

Time-of-flight secondary ion mass spectrometry (TOF-SIMS) emerges as a promising tool to identify the ions (small molecules) indicative of disease states from the surface of patient tissues. In TOF-SIMS analysis, an enhanced ionization of surface molecules is critical to increase the number of detected ions. Several methods have been developed to enhance ionization capability. However, how these methods improve identification of disease-related ions has not been systematically explored. Here, we present a multi-dimensional SIMS (MD-SIMS) that combines conventional TOF-SIMS and metal-assisted SIMS (MetA-SIMS). Using this approach, we analyzed cancer and adjacent normal tissues first by TOF-SIMS and subsequently by MetA-SIMS. In total, TOF- and MetA-SIMS detected 632 and 959 ions, respectively. Among them, 426 were commonly detected by both methods, while 206 and 533 were detected uniquely by TOF- and MetA-SIMS, respectively. Of the 426 commonly detected ions, 250 increased in their intensities by MetA-SIMS, whereas 176 decreased. The integrated analysis of the ions detected by the two methods resulted in an increased number of discriminatory ions leading to an enhanced separation between cancer and normal tissues. Therefore, the results show that MD-SIMS can be a useful approach to provide a comprehensive list of discriminatory ions indicative of disease states.

Time-of-flight secondary ion mass spectrometry (TOF-SIMS) has been used to study surface properties of specimens due to its high molecular specificity and surface sensitivity^1^,^2^,^3^. TOF-SIMS spectra represent chemical compositions of positive and negative secondary ions (small molecules) in the very near surface region. Recently, TOF-SIMS has been used to study the surface of complex biological samples such as cells^4^,^5^,^6^ and tissues^7^,^8^,^9^,^10^. Furthermore, TOF-SIMS can be used to characterize secondary ions from the surface of tissue samples. Comparative analysis of the detected ions between disease and normal samples can reveal the ions indicative of disease states^4^,^9^. For example, Yun *et al.*^9^ catalogued secondary ions from seven gastric cancer and eight normal tissues using TOF-SIMS and found 66 ions discriminating gastric cancer tissues from normal ones. Such studies show that TOF-SIMS is emerging as a promising tool to identify the ions that reflect disease states from the surface of patient tissues.

In TOF-SIMS analysis, enhancing the ionization of small molecules from the surface of a specimen is important to increase the number of detected ions, and several methods have been developed to improve ionization capability. First, Wu *et al.*^11^ proposed a matrix-enhanced SIMS analysis where a matrix (2,5-dihydroxybenzoic acid) used commonly in matrix assisted laser desorption ionization (MALDI) is deposited on the surface of the specimen, followed by TOF-SIMS analysis. In this analysis, ion-beam sputtering of the solid mixtures of analytes and matrix resulted in an increased production of high mass ions. Second, Linton *et al.*^12^ introduced metal-assisted SIMS (MetA-SIMS) analysis where Ag is deposited on the low-density poly(ethylene) surface, followed by TOF-SIMS analysis. They found that Ag deposition resulted in an increased emission of secondary ions from the surface. Recently, several studies^13^,^14^,^15^ have further demonstrated that submonolayer depositions of silver or gold on the surface improve the ionization of surface molecules. However, how these methods increase the number of detected ions and thereby improve the efficiency for identifying disease-related ions has not been systematically investigated.

Here, we present a multi-dimensional TOF-SIMS (MD-SIMS) approach for effective profiling of the secondary ions from the surface of tissue samples, which can lead to an efficient identification of disease-related ions at the tissue surface. This approach first uses conventional TOF-SIMS to analyze the fresh surface of the tissues, after which Au is deposited on the surface, followed by TOF-SIMS analysis (i.e., MetA-SIMS). This serial analysis provides multi-dimensional data generated by both the conventional TOF-SIMS and MetA-SIMS. To demonstrate the utility of a MD-SIMS analysis, we analyzed ovarian cancer and adjacent normal tissues sequentially using TOF- and MetA-SIMS. The comparison of the TOF- and MetA-SIMS data revealed shared and distinct ions detected by the two SIMS methods, indicating that particular signals were suppressed and enhanced by MetA-SIMS. By combining the ions detected by TOF- and MetA-SIMS, the MD-SIMS analysis produced an increased number of discriminatory ions between cancer and normal tissues, which can be used as indicators of disease states.

## Results

### MD-SIMS analysis of ovarian cancer and normal tissues

**Figure 1:**
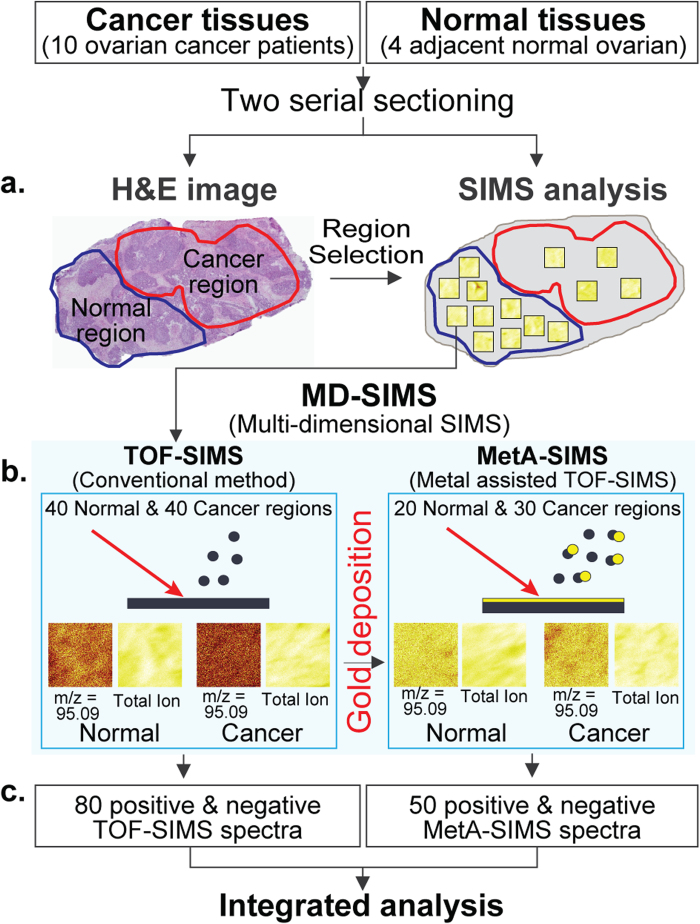
Overview of the MD-SIMS framework.

For a comparative analysis of the secondary ions at the tissue surface, we collected the tissues from 10 ovarian cancer patients. The characteristics of the patients are summarized in Supplementary Table S1. For each of the 10 ovarian tissues, we first performed two serial sectioning. One section was used for H&E staining to identify cancer and adjacent normal regions, whereas the other section was used for TOF- and MetA-SIMS analyses (Fig. 1a). Based on the H&E staining, we selected 40 different cancer and adjacent normal regions (e.g. boxes in Fig. 1a) that contain high densities of cancer and adjacent normal cells, respectively, from the 10 ovarian cancer tissues (**Methods**). Multiple areas in each tissue were analyzed to account for the heterogeneity arisen from the varying amounts of cancer cells (see **Discussion**). For each of the 80 selected regions, we performed TOF-SIMS analysis (Fig. 1b, left box). Subsequently, we performed MetA-SIMS analysis after depositing Au on the same regions for which TOF-SIMS analysis was performed (Fig. 1b, right box). The MetA-SIMS analysis was performed for 50 of the 80 selected regions (30 cancer and 20 adjacent normal regions). In each SIMS analysis, we analyzed the secondary ions with both positive and negative modes. Thus, these analyses resulted in 80 positive and negative TOF-SIMS spectra and 50 positive and negative MetA-SIMS spectra (Fig. 1c).

### Comparison of TOF-SIMS with MetA-SIMS data

**Figure 2:**
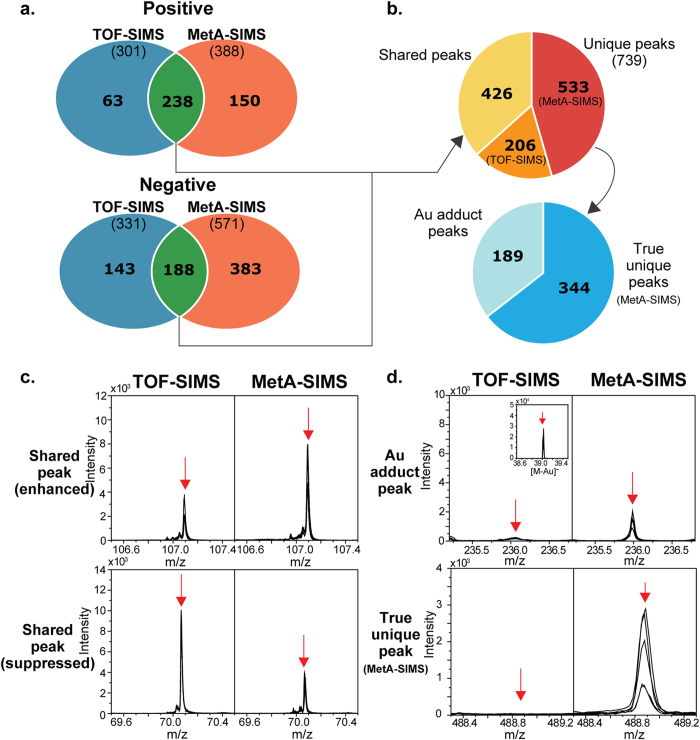
Shared and distinct ions detected by TOF-SIMS and MetA-SIMS analyses. (**a**). Relationships among positive (top) or negative (bottom) peaks measured by TOF-SIMS and MetA-SIMS analyses. (**b**). Proportion of the peaks commonly (shared) and uniquely (unique) detected by TOF-SIMS and MetA-SIMS analyses. The second pie chart shows the composition of the peaks uniquely detected by MetA-SIMS analysis. (**c**). Examples of the peaks detected commonly by TOF- and MetA-SIMS analyses with enhanced and suppressed ionizations in MetA-SIMS analysis, compared to those in TOF-SIMS analysis. (**d**). Examples of an Au adduct peak and a peak uniquely detected by MetA-SIMS analysis.

For each dataset (80 TOF-SIMS spectra or 50 MetA-SIMS spectra), we identified the ion peaks and then aligned the peaks using TOFSIMS-P as previously described^9^. Of these ion peaks, we focused on the peaks that were detected in more than 50% of ovarian cancer and normal tissue samples in each dataset (i.e., 20 cancer and 20 normal samples in TOF-SIMS datasets; 15 cancer and 10 normal samples in MetA-SIMS dataset), as previously reported^9^. The 50% cutoff was used to ensure statistical reliability in the following analyses of the peaks including the comparison of TOF- and MetA-SIMS and the identification of discriminatory ions between ovarian cancer and normal tissues (see **Discussion**). Based on this criterion, we identified 301 positive and 331 negative ion peaks from the 80 TOF-SIMS spectra, and 388 positive and 571 negative ion peaks from the 50 MetA-SIMS spectra (Fig. 2a).

Of these peaks, 426 were detected commonly by both TOF- and MetA-SIMS analyses (Fig. 2b, 1^st^ pie chart). Of the 426 shared peaks, 250 were increased in their intensities by MetA-SIMS analysis, compared to the TOF-SIMS analysis, indicating enhanced ionization of the corresponding ions by Au deposition (Fig. 2c, top). In contrast, 176 showed decreased intensities in MetA-SIMS analysis (Fig. 2c, bottom). This suggests that Au deposition can also suppress the ionization of these ions. On the other hand, 739 were detected uniquely by TOF-SIMS (206 peaks) or MetA-SIMS analysis (533 peaks), respectively (Fig. 2b, 1^st^ pie chart). The 533 peaks uniquely detected by MetA-SIMS analysis comprise 1) Au adducts (189 peaks) for which the same ions were detected at m/z-196.97 (Au) by TOF-SIMS analysis (Fig. 2d, top) and 2) the 344 peaks detected truly uniquely by MetA-SIMS analysis (Fig. 2d, bottom) (Fig. 2b, 2^nd^ pie chart). These data indicate that the two SIMS analyses provide complementary information for the compositions of the secondary ions from the tissue surface by detecting shared and distinct ions with different intensities.

### Discriminatory ions between ovarian cancer and normal tissues

**Figure 3:**
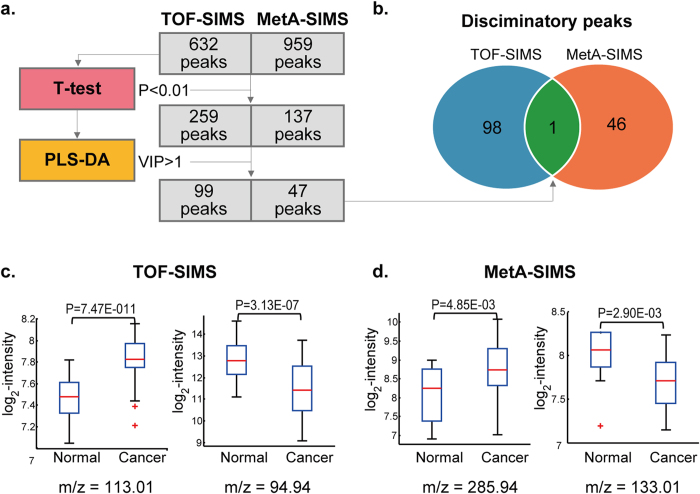
Analysis of TOF- and MetA-SIMS datasets. (**a**). Analytical framework for identification of discriminatory ions. (**b**). Relationships between the discriminatory ions identified from TOF- and MetA-SIMS datasets. (**c**–**d**). Up- or down-regulation of four example discriminatory ions in ovarian cancer tissues detected by TOF- (**c**) and MetA-SIMS analyses (**d**), compared to normal tissues.

To identify the ions indicative of disease states, we next identified the peaks that discriminated the ovarian cancer samples from the normal samples. To this end, we first combined positive and negative ion peaks detected from TOF- and MetA-SIMS analyses. From all of these detected peaks, we used only the 632 and 959 peaks in the TOF- and MetA-SIMS datasets, respectively, (Fig. 2a), which were detected in more than 50% of ovarian cancer and normal tissue samples to ensure their statistical power as discriminatory peaks (see **Discussion**). We then applied two-tailed T-test and also partial least square-discriminant analysis (PLS-DA) to these peaks. Of them, we finally selected 99 discriminatory peaks from TOF-SIMS data and 47 discriminatory peaks from MetA-SIMS data with P < 0.01 using two-tailed T-test and VIP > 1 obtained from PLS-DA, as previously described^9^ (Fig. 3a; Supplementary Table S2; **Methods**). Among these discriminatory peaks, only one was identified commonly from the TOF- and MetA-SIMS data, while 98 and 46 were identified uniquely in TOF- and MetA-SIMS data, respectively (Fig. 3b). The uniquely identified discriminatory peaks indicate that MD-SIMS analysis can provide a more comprehensive view of the discriminatory signatures between ovarian cancer and normal samples than a single analysis of TOF- or MetA-SIMS. Of the 145 discriminatory peaks, four example peaks showing up- or down-regulated in ovarian cancer tissues, compared to adjacent normal tissues, are shown in Fig. 3c,d.

### Comprehensive signatures to enhance discrimination between ovarian cancer and normal tissues

**Figure 4:**
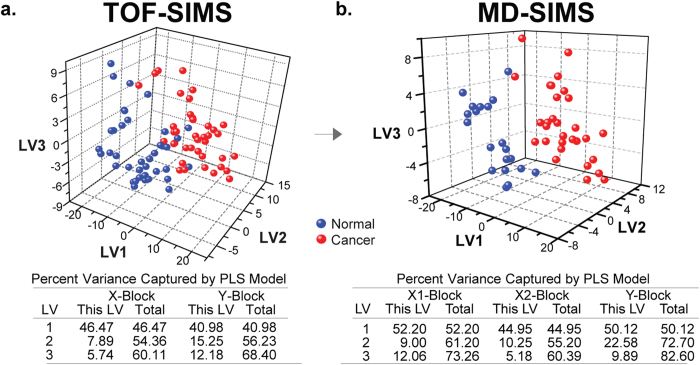
Integrated analysis of TOF- and MetA-SIMS datasets. 3-d PLS-DA score plots for TOF-SIMS (**a**) and MD-SIMS (**b**). LV1-3, 1^st^ to 3^rd^ PLS latent variables (LVs). The tables show that MD-SIMS enhanced the power of discrimination of ovarian cancer samples (red) from normal samples (blue), compared to TOF-SIMS. X-block, intensities of the 99 discriminatory peaks in TOF-SIMS data; X1- and X2-blocks, intensities of the 145 discriminatory peaks in TOF- and MetA-SIMS data, respectively; Y-block, binary vector in which ones and zeros represent ovarian cancer and normal samples, respectively. A percent variance captured by an LV in X- or Y-blocks indicates the amount of the information in X-block used to explain the amount of the separation explained by the LV among the binary values in Y-block.

Compared to the conventional TOF-SIMS analysis, the additional MetA-SIMS analysis in MD-SIMS analysis increased the number of discriminatory ions (Fig. 3b). The extended list of the discriminatory peaks can provide a better discrimination between ovarian cancer and adjacent normal tissues. To test this notion, we applied PLS-DA to the 99 discriminatory peaks (Fig. 3b) identified by the conventional TOF-SIMS analysis and also multi-block PLS-DA (MPLS-DA) to the 145 peaks (Fig. 3b) identified by MD-SIMS analysis. PLS-DA showed that 68.4% of the separation between ovarian cancer and normal samples was achieved by the 99 discriminatory peaks identified by TOF-SIMS analysis. With these 99 peaks, a partial overlap between the ovarian cancer and normal samples was observed in the PLS latent space (Fig. 4a). In contrast, MPLS-DA showed that the use of the additional discriminatory peaks identified by MD-SIMS analysis improved the separation to 82.6%, resulting in a reduced overlap between the ovarian cancer and normal samples, compared to that observed from PLS-DA using only the discriminatory peaks identified by TOF-SIMS analysis (Fig. 4b). This result indicates that MD-SIMS analysis provided a comprehensive list of molecular signatures that can better discriminate ovarian cancer samples from normal samples.

## Discussion

Although TOF-SIMS is a promising tool to identify the secondary ions reflecting disease states at the surface of patient tissues, its use has been hampered by the limited capacity of detecting the ions. Thus, several methods have been developed to improve the capability to ionize the ions at the surface. This has led to an increased number of the detected secondary ions, but the usefulness of these methods to efficiently identify the ions indicating disease states has not been systematically explored. In this study, we proposed MD-SIMS analysis, a combination of TOF-SIMS and MetA-SIMS analyses. By applying MD-SIMS analysis to ovarian cancer and adjacent normal tissues, we demonstrated that MD-SIMS analysis resulted in a more comprehensive set of the ions associated with disease states, compared to the conventional TOF-SIMS analysis.

A cancer tissue often shows heterogeneous molecular signatures across different regions of the tissue. The variation in the relative amounts of cancer and normal cells across the different regions mainly accounts for the heterogeneity. Different amounts of normal cells in the regions can dilute disease-related signatures with different extents. In this study, given the heterogeneity, to identify the ions that represent disease states, we performed TOF-SIMS analysis in the following manner. First, for each of 10 cancer tissues, we performed TOF-SIMS analyses of 4 different regions (100 × 100 μm^2^ per region) that contain high densities of the cancer cells based on the H&E staining, assuming that the 4 regions can provide sufficient information of cancer-related signatures in each tissue. For comparative analysis, we further performed 10 different regions for each of the 4 cancer tissues containing high densities of adjacent normal cells. These analyses resulted in the 40 cancer and normal datasets. Compared with the numbers of the regions analyzed during the discovery phase in other SIMS and metabolomics studies^9^,^16^,^17^, the 40 cancer regions can be considered to be sufficiently large to provide meaningful cancer-related signatures. Second, we then identified the discriminatory ions as the ones that showed consistent differences in their abundances between the 40 cancer and normal datasets. This helps us focus on identification of the discriminatory ions that can represent the ovarian cancer states.

In this study, we performed MD-SIMS analysis under a static condition (<10^12^ primary ions/cm^2^), assuming that no significant surface damage occurs, as previously reported^18^. Even under this condition, however, organic systems, such as cells or tissues, might be damaged during TOF-SIMS, and MetA-SIMS spectra can include the ions resulting from such damage, thus affecting the accuracy of the MD-SIMS analysis. To examine this issue, we performed two serial TOF-SIMS analyses under a static condition (Case 1) for a region of an ovarian tissue (100 × 100 μm^2^). In the MD-SIMS analysis, for a region of an ovarian tissue, we performed TOF-SIMS analysis followed by MetA-SIMS analysis (Case 2). In Case 1, high correlations (correlation coefficients close to 1) were observed between the intensities of the peaks detected by two serial TOF-SIMS analyses (Supplementary Fig. S1a), indicating that despite potential tissue damage, two serial TOF-SIMS analyses generated highly reproducible sets of the detected ions. In contrast, in Case 2, relatively smaller correlations (correlation coefficients = 0.771 and 0.717 for positive and negative modes, respectively) were observed between the intensities of the peaks detected by TOF- and MetA-SIMS analyses (Supplementary Fig. S1b), indicating that despite potential tissue damage, MetA-SIMS analysis could generate a reasonable level of the peaks independent of those detected by the initial TOF-SIMS analysis.

To identify discriminatory ions, in this study, we used the peaks detected in more than 50% of ovarian cancer and normal tissue samples. The peaks less frequently observed could be either chemical noises arisen from contamination or the peaks for real secondary ions that existed in a small number of the regions or tissues analyzed. The discriminatory peaks observed in a small number of samples can be also meaningful. In this study, however, among the detected peaks, we focused on the peaks that were detected in more than a certain percentage of ovarian cancer and normal tissue samples in each dataset, as previously reported^9^, which can ensure statistically reliable comparison of TOF- and MetA-SIMS and identification of statistically reliable discriminatory ions that are likely to be detected in new tissues by MD-SIMS. To determine the percentage cutoff, we varied the cutoff from 10% to 90% and found, as expected, that a high percentage cutoff decreases the number of the peaks analyzed, increasing the false negative rate in identification of discriminatory ions, whereas a small cutoff increases the number of the peaks, increasing the false positive rate (Supplementary Fig. S2). These data revealed no evidence that a particular percentage cutoff should be used. Thus, we chose 50% as a percentage cutoff such that the conclusions from the following analyses are likely to be valid at least in half of the samples to be analyzed.

Few approaches have been proposed to identify the molecules for the ions detected by TOF-SIMS^19^,^20^,^21^, which often require significant amounts of efforts and time. In this study, our primary goal was to propose MD-SIMS to effectively extend the list of the disease-related ions, rather than to demonstrate biological meanings of these ions by mapping them into molecules and pathways. Thus, we performed no experiments to identify the 145 discriminatory ions. However, it would be important to assign molecules to the discriminatory ions in order to assess whether the extended list of the discriminatory ions by MD-SIMS would be useful. We thus employed a previously reported approach that compares the masses of the 145 discriminatory ions with those of the TOF-SIMS ions for which the molecules were previously identified using the experimental approaches mentioned above^22^. As a result, we identified the molecules for 22 of the 145 discriminatory ions (Supplementary Table S3), including fatty acids (FAs), cholesterol (CH), glycerophospholipids (GP), and phosphatidylethanolamines (PE). CH, PE, and GP were identified from TOF-SIMS, and FAs were additionally identified from MetA-SIMS, showing that the integrated MD-SIMS analysis provided a more comprehensive set of molecules altered in ovarian cancer.

Based on the molecules assigned for the 22 discriminatory ions, we further built a pathway model describing altered lipid metabolism in ovarian cancer (Supplementary Fig. S3). In this model, GP and PE were increased in ovarian cancer tissues, compared to adjacent normal tissues, whereas FA and CH were decreased. Several of these changes have been previously reported to be associated with ovarian cancers. For example, altered expression of enzymes [FA synthase (FASN)^23^, stearoyl-CoA desaturase-1 (SCD1)^24^, and lysophosphatidic acid acyltransferase-β (LPAAT-β)^25^] involved in the GP synthetic pathway was proposed as a potential indicator of the poor clinical outcome of ovarian cancer patients. Also, altered expression of HMG-CoAR(HMGCR), which regulates cholesterol synthesis, was proposed as a predictor of recurrence free survival in ovarian cancer^26^. Moreover, in various cancers, GP or PE, components of plasma membrane, are increased during cell proliferation^27^,^28^, consistent with up-regulation of GP or PE in ovarian cancer detected by MD-SIMS. All these data support potential validity of the extended discriminatory ions by MD-SIMS. However, detailed functional studies should be performed to demonstrate the validity of the discriminatory ions, together with validation of the discriminatory ions in large-scale cohorts. Furthermore, only a limited number of ovarian cancer samples (n = 10) was used in this study. Thus, the validity of the discriminatory ions should be tested in large-scale validation experiments, together with the detailed functional studies.

Principal component analysis (PCA) has been applied to the TOF-SIMS data to effectively explore the data structure in an unsupervised manner^29^. In this study, however, we only applied PLS-DA to select the discriminatory ions based on the VIPs of the ions, together with the T-test. Thus, to examine whether PCA can provide additional knowledge of the data structure, we performed PCA using the 1,591 ion peaks (632 from TOF-SIMS and 959 from MetA-SIMS) to which PLS-DA was applied to select the discriminatory ions based on their VIPs. The first three PCs captured 54.5% (22.22, 18.97, and 13.30% respectively) of the total variance in the data (Supplementary Fig. S4a). PC1 (22.22%) showed no correlation with the difference between ovarian cancer and normal tissue samples, suggesting that the data contained a high level of variability independent of the difference between cancer and normal samples. However, PC2 (18.97%) began to show a significant (P = 4.77 × 10^−4^) correlation with such difference, indicating that the data also contained significant amount of information regarding the difference between cancer and normal samples (Supplementary Fig. S4b). To understand how PLS-DA handles these variabilities in the data independent of the difference between cancer and normal samples, we applied multi-block PLS-DA to the same data (Supplementary Fig. S4c). The comparison of both PCA and PLS-DA results showed that the application of PLS-DA to the 1,591 ion peaks allowed us to effectively focus on the information relevant to the difference between cancer and normal samples, thereby enabling correct identification of the discriminatory ions based on the VIPs. Moreover, multi-block PLS-DA further showed how the discriminatory ions identified from both TOF- and MetA-SIMS data additively contributed to capturing the difference between ovarian cancer and normal samples. In summary, MD-SIMS can be a useful approach to provide a more comprehensive list of the discriminatory ions that can be used to understand the disease states.

## Methods

### Sample preparation

Tissues were obtained from 10 ovarian cancer patients hospitalized at the National Cancer Center in Korea from 2005 to 2008 with informed consent in accordance with the guidelines approved by the institutional review board at the National Cancer Center (approval no. NCCNCS 09-309)^30^. Samples were rapidly frozen in liquid nitrogen upon acquisition and then stored at −80 °C before TOF-SIMS analysis. The samples were processed for TOF-SIMS analysis as previously described^31^. Briefly, two serial sections of each tissue of 10 μm-thickness were generated at −20°C using a cryostat (Leica CM 3050S, Leica Microsystems Inc., IL). The 1^st^ section was affixed to a glass slide and stained with hematoxylin and eosin (H&E) to identify the areas enriched with normal or cancer cells. The 2^nd^ section was deposited onto a Si wafer that was sonicated with ethanol and acetone for 5 min each, rinsed with water, and then stored at −80 °C until TOF-SIMS analysis. No chemical fixation was done because of the possibility that chemical artefacts could be detected by TOF-SIMS. Based on the H&E staining, we selected 4 different regions containing high densities of cancer cells from the 2nd section for each of the 10 ovarian cancer tissues (40 cancer regions). Also, we selected 40 adjacent normal regions from the ovarian tissues. To this end, we first selected 4 of 10 tissues containing high densities of adjacent normal cells, based on the H&E staining, and then further selected 10 adjacent normal regions from each of the 4 ovarian tissues (40 adjacent normal regions). Each selected region was analyzed by TOF-SIMS in both the positive and negative modes. After the TOF-SIMS analysis, Au was deposited onto the tissue surface using a Quorum Technologies (Newhaven, East Sussex, U.K.) SC7640 sputter coater. The thickness of the gold was controlled to be 1 nm by a FT7607 quartz crystal microbalance stage and a FT7690 film thickness monitor. The samples were then analyzed by TOF-SIMS after metal deposition.

### TOF-SIMS analysis

We used a TOF-SIMS V instrument (ION-TOF GmbH, Germany) equipped with a bismuth liquid metal ion gun (LMIG). A Bi_3_^+^ primary beam at 25 keV in a high-current bunched mode (pulse width = 0.65 ns and beam diameter = 3 μm) with a target current of 0.2 pA and a repetition rate of 5 kHz was used to obtain positive and negative spectra. The analysis area of 100 × 100 μm^2^ (128 × 128 pixels) was randomly rastered by the primary ions and were charge-compensated for the tissue samples by low-energy electron flooding. The primary ion dose density was maintained below 10^12^ ions/cm^2^ to ensure a static SIMS condition. Mass resolution was higher than 7000 at m/z < 500 in both the positive and negative modes. Positive and negative ion spectra from each sample at specific areas were obtained. Positive and negative ion spectra were internally mass calibrated using CH_3_^+^, C_2_H_3_^+^, C_3_H_5_^+^ and C_5_H_14_NO^+^ peaks, and CH^−^, C_2_H^−^, C_4_H^−^ and C_18_H_35_O_2_^−^ peaks, respectively. After the calibration, the resulting mass accuracy was 60 ppm for m/z < 200 and 150 ppm for m/z > 200 on average. Each spectrum was exported as an ASCII file.

### Analysis of TOF-SIMS or MetA-SIMS data

From each of the spectra generated by TOF-SIMS, the peaks were automatically identified using the TOFBAT program in the built-in IonSpec software from ION-TOF. We searched for those peaks whose intensities were larger than 20 between 1 and 800 m/z. For each peak, the m/z, m/z range, and area of the peak were identified. Using the peaks from all the samples, we then applied a computational platform, TOFSIMS-P^9^, as previously reported for comparative analysis of the TOF-SIMS data obtained from cancer and normal samples, which involved 1) refinement and alignment of the peaks identified from all the samples and 2) selection of discriminatory ions. The same procedure was used to analyze the MetA-SIMS dataset.

### Partial least square-discriminant analysis (PLS-DA)

PLS-DA was applied to the combined set of positive and negative ion peaks detected from TOF-SIMS or MetA-SIMS analysis, as previously described^9^. Briefly, we defined X-block with the intensities of *k* peaks in *n* samples (i.e. *n*  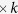 alignment matrix) and *n*  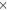 1 Y-block with information of sample classes (one for cancer and zero for normal). First, we estimated the missing values (less than 50% of intensities in the alignment table) using the k-nearest-neighbor imputing method^32^. Second, we selected the number of latent variables (LVs) as the one for which the mean error rate, computed by performing 10-fold cross-validations (CVs) 1000 times, reached the minimum as previously described^9^. Finally, we calculated the mean of variable importance on the projection (VIP) values for each peak by performing the 10-fold CV 1000 times using the determined number of PLS LVs. We selected the discriminatory ion peaks as the ones with the mean VIP > 1^33^,^34^.

## Additional Information

**How to cite this article**: Park, J.-W. *et al.* Multi-dimensional TOF-SIMS analysis for effective profiling of disease-related ions from the tissue surface. *Sci. Rep.* **5**, 11077; doi: 10.1038/srep11077 (2015).

## Supplementary Material

Supplementary Information

## References

[b1] Belu A. M., Graham D. J. & Castner D. G. Time-of-flight secondary ion mass spectrometry: techniques and applications for the characterization of biomaterial surfaces. Biomaterials 24, 3635–3653 (2003). 12818535 10.1016/s0142-9612(03)00159-5

[b2] Wang H., Castner D. G., Ratner B. D. & Jiang S. Probing the orientation of surface-immobilized immunoglobulin G by time-of-flight secondary ion mass spectrometry. Langmuir 20, 1877–87 (2004). 15801458 10.1021/la035376f

[b3] Benninghoven A. Chemical Analysis of Inorganic and Organic Surfaces and Thin Films by Static Time-of-Flight Secondary Ion Mass Spectrometry (TOF-SIMS). Angew. Chemie Int. Ed. English 33, 1023–1043 (1994).

[b4] Kulp K. S. *et al.* Chemical and biological differentiation of three human breast cancer cell types using time-of-flight secondary ion mass spectrometry. Anal. Chem. 78, 3651–3658 (2006). 16737220 10.1021/ac060054c

[b5] Nygren K. A. N., Hagenhoff B., Malmberg P. E. R., Nilsson M. & Richter K. Bioimaging TOF-SIMS: High Resolution 3D Imaging of Single Cells. Microsc. Res. Tech. 974, 969–974 (2007). 17661396 10.1002/jemt.20502

[b6] Fletcher J. *et al.* A new dynamic in mass spectral imaging of single biological cells. Anal. Chem. 80, 9058–9064 (2008). 19551933 10.1021/ac8015278

[b7] Touboul D., Halgand F. & Brunelle A. Tissue molecular ion imaging by gold cluster ion bombardment. Anal. Chem 76, 1550–1559 (2004). 15018551 10.1021/ac035243z

[b8] Sjövall P., Johansson B. & Lausmaa J. Localization of lipids in freeze-dried mouse brain sections by imaging TOF-SIMS. Appl. Surf. Sci. 252, 6966–6974 (2006).

[b9] Yun S. *et al.* TOFSIMS-P: A web-based platform for analysis of large-scale TOF-SIMS data. Anal. Chem. 83, 9298–305 (2011). 22054246 10.1021/ac2016932

[b10] McDonnell L. a *et al.* Subcellular imaging mass spectrometry of brain tissue. J. Mass Spectrom. 40, 160–8 (2005). 15706616 10.1002/jms.735

[b11] Wu K. J. & Odom R. W. Matrix-enhanced secondary ion mass spectrometry: a method for molecular analysis of solid surfaces. Anal. Chem. 68, 873–82 (1996). 21619183 10.1021/ac950717i

[b12] Linton R. W. *et al.* Time-of-flight secondary ion mass spectrometric analysis of polymer surfaces and additives. Surf. Interface Anal. 20, 991–999 (1993).

[b13] Delcorte a. *et al.* Metal-Assisted Secondary Ion Mass Spectrometry Using Atomic (Ga + , In +) and Fullerene Projectiles. Anal. Chem. 79, 3673–3689 (2007). 17417819 10.1021/ac062406l

[b14] Altelaar A., Klinkert I. & Jalink K. Gold-enhanced biomolecular surface imaging of cells and tissue by SIMS and MALDI mass spectrometry. Anal. Chem 78, 734–42 (2006). 16448046 10.1021/ac0513111

[b15] Adriaensen L., Vangaever F. & Gijbels R. Metal-assisted secondary ion mass spectrometry: influence of Ag and Au deposition on molecular ion yields. Anal. Chem. 76, 6777–85 (2004). 15538803 10.1021/ac049108d

[b16] Hansson M. *et al.* Iodine content and distribution in extratumoral and tumor thyroid tissue analyzed with X-ray fluorescence and time-of-flight secondary ion mass spectrometry. Thyroid 18, 1215–20 (2008). 19014328 10.1089/thy.2008.0020

[b17] Veselkov K. a *et al.* Chemo-informatic strategy for imaging mass spectrometry-based hyperspectral profiling of lipid signatures in colorectal cancer. Proc. Natl. Acad. Sci. U. S. A. 111, 1216–21 (2014). 24398526 10.1073/pnas.1310524111 PMC3903245

[b18] Vickerman J. C., Briggs J. C. V. D. & Briggs D. in ToF-SIMS: Materials Analysis by Mass Spectrometry 2nd edn, Ch. 1, 1–8 (IM Publications, 2013).

[b19] Sjövall P., Lausmaa J. & Johansson B. Mass spectrometric imaging of lipids in brain tissue. Anal. Chem. 76, 4271–4278 (2004). 15283560 10.1021/ac049389p

[b20] Touboul D., Brunelle A. & Laprévote O. Structural analysis of secondary ions by post-source decay in time-of-flight secondary ion mass spectrometry. Rapid Commun. Mass Spectrom. 20, 703–709 (2006). 16447144 10.1002/rcm.2362

[b21] Piehowski P. D. *et al.* MS/MS methodology to improve subcellular mapping of cholesterol using TOF-SIMS. Anal. Chem. 80, 8662–8667 (2008). 18925746 10.1021/ac801591r PMC2597061

[b22] Hanrieder J. & Ewing A. G. Spatial Elucidation of Spinal Cord Lipid- and Metabolite- Regulations in Amyotrophic Lateral Sclerosis. Sci. Rep. 4, 1–7 (2014). 10.1038/srep05266 PMC4053717 24919836

[b23] Wang H. Q. *et al.* Positive feedback regulation between AKT activation and fatty acid synthase expression in ovarian carcinoma cells. Oncogene 24, 3574–3582 (2005). 15806173 10.1038/sj.onc.1208463

[b24] Roongta U. V. *et al.* Cancer Cell Dependence on Unsaturated Fatty Acids Implicates Stearoyl-CoA Desaturase as a Target for Cancer Therapy. Mol. Cancer Res. 9, 1551–1561 (2011). 21954435 10.1158/1541-7786.MCR-11-0126

[b25] Diefenbach C. S. M. *et al.* Lysophosphatidic acid acyltransferase-beta (LPAAT-beta) is highly expressed in advanced ovarian cancer and is associated with aggressive histology and poor survival. Cancer 107, 1511–1519 (2006). 16944535 10.1002/cncr.22184

[b26] Brennan D. J. *et al.* Tumour-specific HMG-CoAR is an independent predictor of recurrence free survival in epithelial ovarian cancer. BMC Cancer 10, 125 (2010). 20359358 10.1186/1471-2407-10-125 PMC3087316

[b27] DeBerardinis R. J., Lum J. J., Hatzivassiliou G. & Thompson C. B. The biology of cancer: metabolic reprogramming fuels cell growth and proliferation. Cell Metab. 7, 11–20 (2008). 18177721 10.1016/j.cmet.2007.10.002

[b28] Currie E., Schulze A., Zechner R., Walther T. C. & Farese R. V. Cellular fatty acid metabolism and cancer. Cell Metab. 18, 153–161 (2013). 23791484 10.1016/j.cmet.2013.05.017 PMC3742569

[b29] Worley B. & Powers R. Multivariate Analysis in Metabolomics. Curr. Metabolomics 1, 92–107 (2013). 10.2174/2213235X11301010092 PMC4465187 26078916

[b30] Kang S., Lee A., Park Y. & Lee S. Alteration in lipid and protein profiles of ovarian cancer: similarity to breast cancer. Int. J. Gynecol. Cancer 21, 1566–72 (2011). 22123712 10.1097/IGC.0b013e318226c5f5

[b31] Brunelle A., Touboul D. & Laprévote O. Biological tissue imaging with time-of-flight secondary ion mass spectrometry and cluster ion sources. J. Mass Spectrom. 40, 985–99 (2005). 16106340 10.1002/jms.902

[b32] Albrecht D., Kniemeyer O., Brakhage A. a & Guthke R. Missing values in gel-based proteomics. Proteomics 10, 1202–11 (2010). 20077407 10.1002/pmic.200800576

[b33] Chan C., Hwang D., Stephanopoulos G. N., Yarmush M. L. & Stephanopoulos G. Application of multivariate analysis to optimize function of cultured hepatocytes. Biotechnol. Prog. 19, 580–98 (2003). 12675604 10.1021/bp025660h

[b34] Chong I.-G. & Jun C.-H. Performance of some variable selection methods when multicollinearity is present. Chemom. Intell. Lab. Syst. 78, 103–112 (2005).

