# Supplementary material for: Multi-dimensional TOF-SIMS analysis for effective profiling of disease-related ions from the tissue surface
Source: Sci Rep. 2015 Jun 5;5:11077. doi: 10.1038/srep11077 (PMC4457153; doi:10.1038/srep11077)
Supplement: Supplementary Information [file srep11077-s1.pdf]

Supplementary Information

Multi-dimensional TOF-SIMS analysis for effective profiling of disease-related ions from the tissue surface

Ji-Won Park[1,2,7,#], *Hyobin Jeong*[3,#], *Byeongsoo Kang*[4], *Su Jin Kim*[1,5], *Sang Yoon Park*[6], *Sokbom Kang*[6], *Hark Kyun Kim*[6], *Joon Sig Choi*[5], *Daehee Hwang*[3,4,7,*], *Tae Geol Lee*[1,2,*]

[1]Center for Nano-Bio Measurement, Korea Research Institute of Standards and Science, Daejeon, Republic of Korea; [2]Department of Nano and Bio Surface Science, University of Science and Technology, Daejeon, Republic of Korea; [3]School of interdisciplinary bioscience and bioengineering, POSTECH, Pohang, Republic of Korea; [4]Department of New Biology, DGIST, Daegu, Republic of Korea; [5]Department of Biochemistry, Chungnam National University, Daejeon, Republic of Korea; [6]National Cancer Center, Goyang, Republic of Korea; [7]Center for Systems Biology of Plant Senescence and Life History, Institute for Basic Science, Daegu, Republic of Korea.

Supplementary Figures

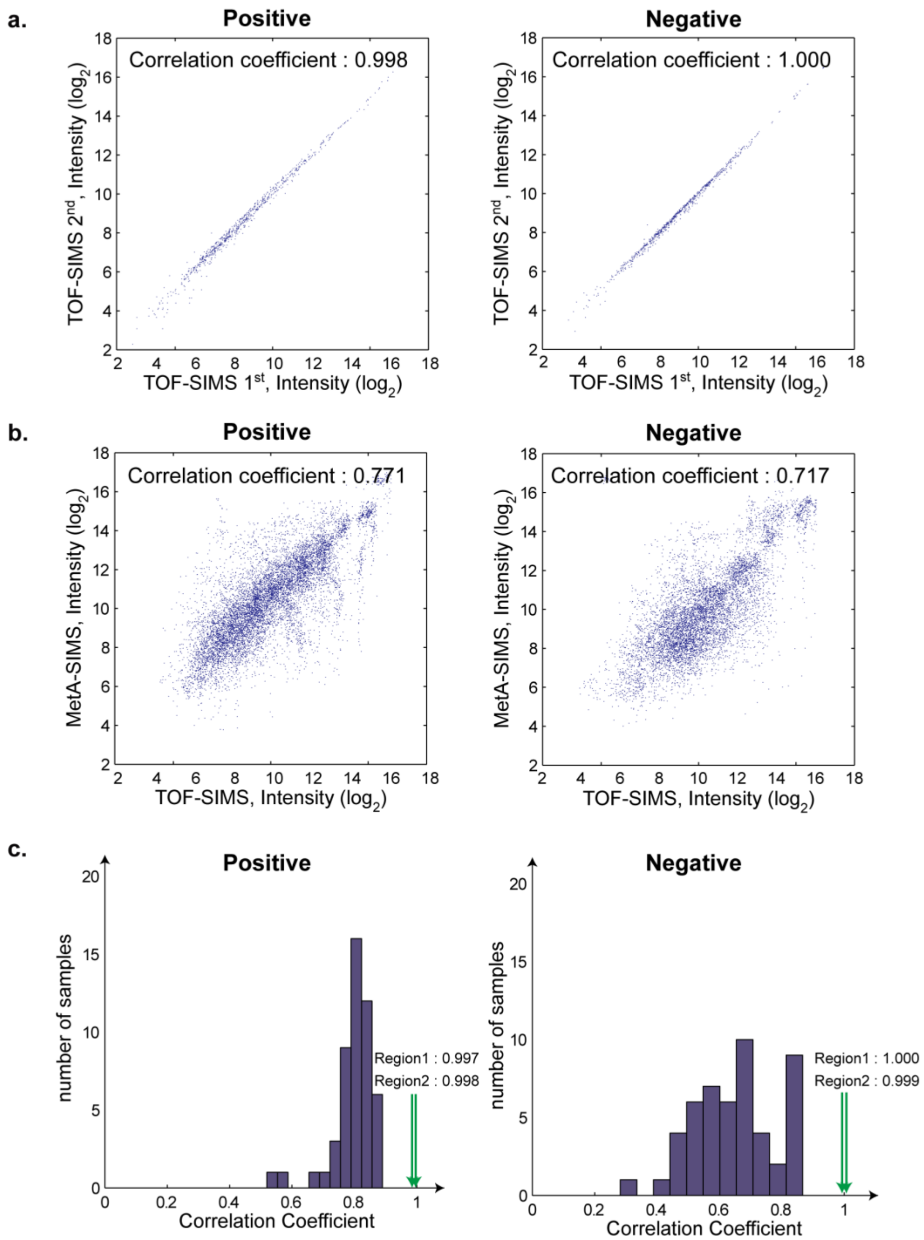

Fig. S1. Correlation analysis of intensities for the peaks detected by two serial TOF-SIMS analyses and also by TOF-SIMS and Meta-SIMS analyses under static conditions.

(a). Scatter plots of intensities of the peaks detected by two serial TOF-SIMS analyses in positive and negative modes (left and right panels, respectively). The data for two different regions were combined for the scatter plot analysis. (b). Scatter plots of intensities of the 426 shared peaks detected by TOF-SIMS and Meta-SIMS analyses in positive and negative modes (left and right panels, respectively). The data obtained for 50 different regions were combined for the scatter plot analysis. (c). Distribution of correlation coefficients for the 50 different regions analyzed by MD-SIMS. For the comparison, the correlation coefficients for the two serial TOF-SIMS analyses for regions 1 and 2 were indicated by the green arrows.

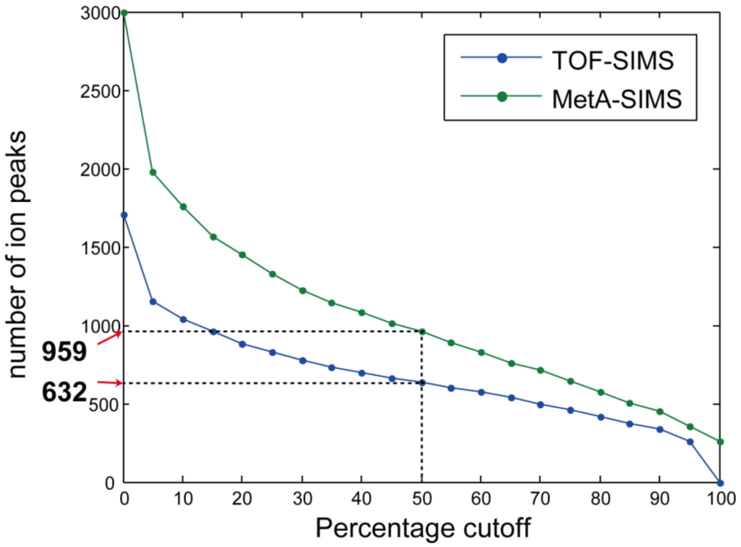

Fig. S2. The numbers of the peaks selected with increasing percentage cutoffs. The numbers of peaks selected from TOF- and MetA-SIMS data with the percentage cutoff used (50%) are indicated in y-axis (arrows and dotted lines).

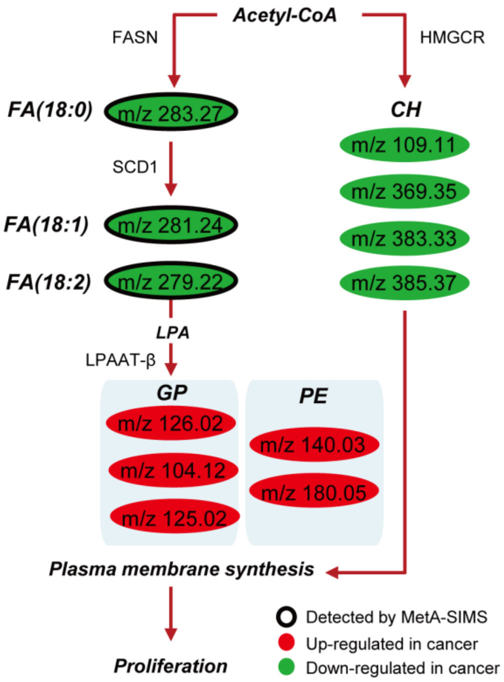

Fig. S3. A pathway model describing dysregulation of lipid metabolism in ovarian cancer. Node colors represent up- (red) and down-regulation (green) of the molecules assigned to the discriminatory ions in ovarian cancer tissues, compared to adjacent normal tissues. Node boundaries (black) indicate the discriminatory ions only detected by MetA-SIMS. FA, fatty acids; CH, cholesterol; FASN, fatty acid synthase; SCD1, stearoyl-CoA desaturase-1; LPAAT-β, lysophosphatidic acid acyltransferase-β; LPA, lysophosphatidic acid; HMGCR, HMG-CoA reductase; GP, Glycerophospholipids; PE, phosphatidylethanolamines.

a.

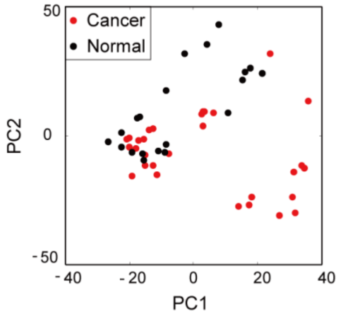

PC#	%Var	%TotVar
1	22.22	22.22
2	18.97	41.19
3	13.30	54.49
4	9.38	63.87
5	5.72	69.59
6	4.53	74.12
7	4.23	78.35
8	2.43	80.79
9	2.09	82.88
10	1.89	84.77

b.

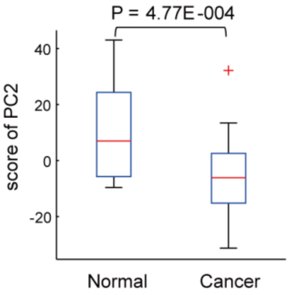

c.

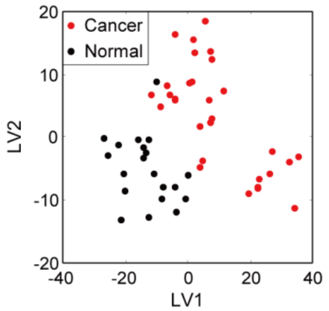

	X1-Block		X2-Block		Y-Block	
LV#	%Var	%TotVar	%Var	%TotVar	%Var	%TotVar
1	25.71	25.71	24.84	24.84	48.10	48.10
2	16.33	42.04	8.93	33.77	27.06	75.16
3	10.44	52.48	26.81	60.58	4.81	79.97
4	8.92	61.39	10.44	71.02	3.82	83.79
5	6.96	68.36	4.74	75.76	6.22	90.01
6	7.33	75.69	2.53	78.29	3.66	93.68
7	4.24	79.92	5.88	84.17	2.31	95.99
8	2.11	82.04	2.40	86.57	1.46	97.45
9	1.43	83.47	1.32	87.89	1.08	98.53
10	2.02	85.48	1.20	89.09	0.40	98.93

Fig. S4. PCA and PLS-DA results of MD-SIMS datasets. (a). 2-d score plot obtained from PCA using the 1,591 ion peaks detected from TOF- and MetA-SIMS. Red and black points indicate ovarian cancer and normal samples, respectively. The table shows explained variances by individual PCs. (b). Boxplot of PC2 scores showing a significant correlation with predefined cancer and normal classes. (c) 2-d score plot obtained from multi-block PLS-DA using the 1,591 ion peaks detected from TOF- and MetA-SIMS for the discrimination of ovarian cancer samples (red) from normal samples (black). The table shows explained variances by individual latent variables (LVs). X1- and X2-blocks, intensities of the 1,591 identified peaks in TOF- and MetA-SIMS data, respectively; Y-block, binary vector in which ones and zeros represent ovarian cancer and normal samples, respectively.

Supplementary Tables

Table S1. Characteristics of the patients.

		Tumor	Normal
Age(years)	Range	25-67	47-66
	Median	51	52
Histologic type	Serous	8(80%)	
	Endometrioid	2(20%)	
Stage (FIGO)	IIC	1(10%)	
	IIIB	1(10%)	
	IIIC	4(40%)	
	IV	4(40%)	
Grade	1	1(10%)	
	2	2(20%)	
	3	7(70%)	
Total No.		10	4

Table S2. Discriminatory peaks. For the discriminatory peaks, the table shows 1) m/zs, 2) up/down-regulation in ovarian cancer measured by MD-SIMS analysis, 3) P-values computed for TOF- and MetA-SIMS data using T-tests, 4) VIPs computed for TOF- and MetA-SIMS data using PLS-DA, 5) whether individual discriminatory peaks were identified from TOF- or MetA-SIMS analysis ('SIMS'), and 6) whether the peaks were observed in the positive or negative mode ('polarity').

m/z	Up/Down-regulation in ovarian cancer measured by MD-SIMS analysis	P-value		VIPs		SIMS		Polarity
		TOF-SIMS	MetA-SIMS	TOF-SIMS	MetA-SIMS	TOF-SIMS	MetA-SIMS	
19.00	Up	0.01	0.01	2.19	1.79	O	O	Negative
19.02	Up	0.00	0.89	1.38	0.00	O	X	Positive
24.00	Down	0.01	0.65	1.16	0.00	O	X	Positive
26.98	Down	-	0.00	0.00	1.92	X	O	Positive
27.02	Up	0.01	0.96	1.59	0.00	O	X	Positive
28.03	Up	0.00	0.68	1.18	0.00	O	X	Positive
29.00	Up	0.00	0.69	1.14	0.00	O	X	Positive
31.02	Down	0.00	0.00	0.83	1.21	X	O	Negative
34.97	Down	0.00	0.45	1.07	0.00	O	X	Negative
36.97	Down	0.00	0.34	1.10	0.00	O	X	Negative
40.03	Up	0.00	0.29	1.34	0.00	O	X	Positive
40.96	Up	-	0.00	0.00	1.09	X	O	Positive
42.01	Up	0.00	0.04	1.27	0.00	O	X	Positive
42.04	Up	0.00	0.96	1.17	0.00	O	X	Positive
44.02	Up	0.00	0.03	1.03	0.00	O	X	Positive
44.05	Up	0.00	0.23	1.18	0.00	O	X	Positive
45.98	Down	0.00	0.95	1.05	0.00	O	X	Positive
46.97	Up	0.00	0.08	1.24	0.00	O	X	Positive
46.99	Down	0.00	0.70	1.01	0.00	O	X	Positive
52.98	Down	0.00	0.40	1.02	0.00	O	X	Negative
54.04	Up	0.00	0.15	1.11	0.00	O	X	Positive
56.97	Up	-	0.00	0.00	1.04	X	O	Positive
57.96	Down	0.00	0.81	1.06	0.00	O	X	Negative
58.01	Down	-	0.00	0.00	1.03	X	O	Negative
58.96	Down	0.00	0.20	1.00	0.00	O	X	Positive
59.02	Down	-	0.00	0.00	1.05	X	O	Negative
59.96	Down	0.00	-	1.06	0.00	O	X	Negative
60.02	Up	0.00	0.06	1.25	0.00	O	X	Positive
62.02	Down	0.00	0.02	1.02	0.00	O	X	Negative
62.98	Down	0.00	0.96	1.04	0.00	O	X	Positive
63.03	Down	-	0.00	0.00	1.14	X	O	Negative
70.95	Down	0.00	0.14	1.09	0.00	O	X	Negative
70.99	Down	0.00	0.32	1.05	0.00	O	X	Positive
71.02	Down	-	0.00	0.00	1.02	X	O	Negative
71.07	Up	0.00	-	1.06	0.00	O	X	Positive
72.08	Up	0.00	0.12	1.08	0.00	O	X	Positive
72.95	Down	0.00	0.26	1.14	0.00	O	X	Negative
73.03	Up	0.00	0.42	1.25	0.00	O	X	Positive
74.94	Down	0.00	0.12	1.97	0.00	O	X	Positive
74.97	Down	0.00	-	1.07	0.00	O	X	Negative
75.03	Down	0.01	-	1.13	0.00	O	X	Negative
75.94	Down	0.00	-	1.89	0.00	O	X	Negative
80.95	Down	0.00	0.74	1.04	0.00	O	X	Positive
82.95	Down	0.00	0.75	1.03	0.00	O	X	Positive
82.98	Down	0.00	0.61	1.05	0.00	O	X	Negative

83.97	Down	0.00	0.63	1.19	0.00	O	X	Negative
84.98	Down	0.00	-	1.01	0.00	O	X	Negative
85.01	Down	-	0.01	0.00	1.25	X	O	Negative
85.97	Down	0.00	0.46	1.12	0.00	O	X	Negative
86.96	Down	0.00	0.42	1.04	0.00	O	X	Positive
87.11	Up	0.00	0.61	1.02	0.00	O	X	Positive
88.08	Up	0.00	0.58	1.02	0.00	O	X	Positive
89.00	Down	-	0.00	0.00	1.11	X	O	Negative
90.95	Down	0.00	0.58	1.07	0.00	O	X	Negative
92.94	Down	0.00	0.26	1.01	0.00	O	X	Negative
94.94	Down	0.00	0.42	1.04	0.00	O	X	Negative
96.09	Down	0.01	0.27	1.27	0.00	O	X	Positive
97.98	Down	0.00	-	1.06	0.00	O	X	Negative
98.98	Down	0.00	-	1.07	0.00	O	X	Negative
98.99	Up	0.00	0.51	1.03	0.00	O	X	Positive
99.95	Down	0.00	-	1.18	0.00	O	X	Negative
99.97	Down	0.00	0.92	1.11	0.00	O	X	Negative
100.01	Down	-	0.00	0.00	1.33	X	O	Negative
100.99	Down	0.00	-	1.07	0.00	O	X	Negative
101.04	Down	-	0.00	0.00	1.21	X	O	Negative
101.96	Down	0.00	0.59	1.03	0.00	O	X	Negative
102.10	Up	0.00	0.14	1.03	0.00	O	X	Positive
103.02	Down	-	0.00	0.00	1.35	X	O	Negative
103.10	Up	0.00	-	1.03	0.00	O	X	Positive
104.12	Up	0.01	-	1.02	0.00	O	X	Positive
105.12	Up	0.00	-	1.26	0.00	O	X	Positive
106.08	Down	0.00	0.42	1.28	0.00	O	X	Positive
107.00	Up	0.00	-	1.12	0.00	O	X	Positive
107.09	Down	0.00	0.12	1.40	0.00	O	X	Positive
109.11	Down	0.01	0.57	1.36	0.00	O	X	Positive
111.14	Down	0.00	-	1.12	0.00	O	X	Positive
112.90	Down	0.00	0.06	1.34	0.00	O	X	Negative
113.01	Up	0.00	-	1.70	0.00	O	X	Positive
114.02	Down	-	0.00	0.00	1.36	X	O	Negative
116.97	Down	-	0.00	0.00	1.06	X	O	Negative
118.09	Down	0.01	0.30	1.21	0.00	O	X	Positive
119.10	Down	0.00	0.14	1.38	0.00	O	X	Positive
121.12	Down	0.00	0.50	1.31	0.00	O	X	Positive
123.14	Down	0.00	0.33	1.25	0.00	O	X	Positive
124.01	Up	0.00	0.22	1.84	0.00	O	X	Negative
125.02	Up	0.00	0.47	1.13	0.00	O	X	Positive
125.96	Down	0.01	-	1.02	0.00	O	X	Positive
126.02	Up	0.00	-	1.07	0.00	O	X	Positive
127.08	Down	-	0.01	0.00	1.11	X	O	Negative
128.97	Down	0.00	-	1.01	0.00	O	X	Negative
130.97	Down	0.00	-	1.02	0.00	O	X	Negative
131.10	Down	0.00	0.98	1.47	0.00	O	X	Positive
132.11	Down	0.00	-	1.28	0.00	O	X	Positive
133.01	Down	-	0.00	0.00	1.21	X	O	Negative
133.12	Down	0.00	0.30	1.33	0.00	O	X	Positive
134.12	Down	0.00	0.15	1.30	0.00	O	X	Positive
137.01	Up	0.00	-	1.04	0.00	O	X	Negative
140.03	Up	0.00	-	1.28	0.00	O	X	Negative

141.93	Up	-	0.01	0.00	1.11	X	O	Negative
143.10	Down	0.01	0.91	1.22	0.00	O	X	Positive
144.10	Down	0.00	0.18	1.29	0.00	O	X	Positive
145.12	Down	0.00	0.17	1.38	0.00	O	X	Positive
146.13	Down	0.00	-	1.25	0.00	O	X	Positive
147.94	Down	0.00	0.32	1.04	0.00	O	X	Positive
148.95	Down	0.00	-	1.01	0.00	O	X	Positive
148.97	Down	-	0.00	0.00	1.10	X	O	Negative
149.16	Down	0.00	-	1.21	0.00	O	X	Positive
150.09	Up	0.00	-	1.07	0.00	O	X	Positive
154.07	Down	0.00	0.69	1.07	0.00	O	X	Positive
158.13	Down	0.00	-	1.13	0.00	O	X	Positive
158.93	Up	0.00	0.00	0.73	1.03	X	O	Positive
159.14	Down	0.00	-	1.38	0.00	O	X	Positive
163.00	Down	-	0.01	0.00	1.04	X	O	Negative
164.94	Down	0.00	-	1.06	0.00	O	X	Positive
170.00	Down	-	0.01	0.00	1.52	X	O	Negative
170.87	Down	0.00	-	1.19	0.00	O	X	Positive
171.00	Down	-	0.00	0.00	1.02	X	O	Negative
171.98	Down	-	0.00	0.00	1.11	X	O	Negative
172.86	Down	0.00	0.72	1.45	0.00	O	X	Positive
173.97	Down	-	0.00	0.00	1.11	X	O	Negative
180.05	Up	0.00	-	1.05	0.00	O	X	Negative
186.99	Down	-	0.00	0.00	1.01	X	O	Negative
189.98	Down	-	0.00	0.00	1.23	X	O	Negative
190.97	Down	-	0.00	0.00	1.02	X	O	Negative
206.98	Down	-	0.00	0.00	1.06	X	O	Negative
207.97	Down	-	0.01	0.00	1.15	X	O	Negative
208.95	Down	-	0.00	0.00	1.09	X	O	Negative
220.97	Down	-	0.01	0.00	1.63	X	O	Positive
222.97	Down	-	0.00	0.00	1.02	X	O	Negative
224.99	Down	-	0.00	0.00	1.17	X	O	Negative
256.23	Down	-	0.00	0.00	1.01	X	O	Negative
276.85	Up	-	0.00	0.00	1.12	X	O	Negative
279.22	Down	-	0.00	0.00	1.06	X	O	Negative
281.24	Down	-	0.00	0.00	1.00	X	O	Negative
283.27	Down	-	0.00	0.00	1.95	X	O	Negative
285.94	Up	-	0.00	0.00	1.05	X	O	Negative
301.93	Up	-	0.00	0.00	1.18	X	O	Negative
344.95	Up	-	0.00	0.00	1.82	X	O	Negative
369.35	Down	0.00	0.79	1.11	0.00	O	X	Positive
383.33	Down	0.00	-	1.28	0.00	O	X	Negative
385.37	Down	0.00	-	1.27	0.00	O	X	Negative
456.87	Up	-	0.00	0.00	1.13	X	O	Negative
469.93	Up	-	0.00	0.00	1.02	X	O	Negative
494.92	Up	-	0.01	0.00	1.00	X	O	Negative
664.87	Up	-	0.00	0.00	1.13	X	O	Negative

Table S3. Molecules assigned for 22 discriminatory ions. The table includes 1) up- or down-regulation of the ions in ovarian cancer tissues, compared to adjacent normal tissues, 2-3) classes and sub-classes of the molecules assigned to the discriminatory ions, 4-5) names and chemical formula of the molecules, 6) whether the ions are protonated or fragments ('Species'), 7) m/zs, 8) whether the corresponding discriminatory peaks were identified from TOF- or MetA-SIMS analysis ('SIMS'), 9) whether the peaks were observed in the positive or negative mode ('polarity') and 10) references where we obtained the information of the TOF-SIMS peaks corresponding to the discriminatory ions.

Cancer/ Normal	Assigned molecules					SIMS result				Reference
	Class	Sub-class	Assignment	Formula	Species	m/z	TOF-SIMS	MetA-SIMS	Polarity	
Up	Glycerophospholipids (GP)	Glycerophosphocholines	GPCho	$C_5H_{14}NO$	Fragment	104.12	O	X	Positive	1
				$C_2H_6PO_4$	Fragment	125.02	O	X	Positive	2
		Glycerophosphoethanolamines	GPEtn	$C_2H_9NPO_3$	Fragment	126.02	O	X	Positive	1
	Phosphatidylethanolamines (PE)	Phosphatidylethanolamine	PE	$C_2H_7NO_4P$	[M]-	140.03	O	X	Negative	3
				$C_5H_{11}NO_4P$	[M]-	180.05	O	X	Negative	3
	Sphingolipids (SP)	-	SP	$C_5H_{12}NO$	[M]+	102.1	O	X	Positive	2
	Prenol Lipids	Quinones and hydroquinones	α-Tocopherol	$C_{10}H_{14}O$	[M]+	150.09	O	X	Positive	4
	Others	-	Protein fragment	CNO	M[CNO−]	42.01	O	X	Positive	5
		-	$C_3H_{10}N_3$	$C_3H_{10}N_3$	[M]+	88.08	O	X	Positive	4
Down	Fatty Acyls (FA)	Fatty acid lipid tail	FA−lipid tail	$C_{10}H_{12}$	[M]+	132.11	O	X	Positive	4
		Unsaturated fatty acid	FA(18:2)	$C_{18}H_{31}O_2$	[M-H]-	279.22	X	O	Negative	1
		Fatty acids and conjugates	FA(18:1)	$C_{18}H_{33}O_2$	[M-H]-	281.24	X	O	Negative	1, 3
		Straight chain fatty acids	FA(18:0)	$C_{18}H_{35}O_2$	[M-H]-	283.27	X	O	Negative	1, 3
	Sterol Lipids (CH)	Cholesterol and derivatives	CH	C_8H_{13}	Fragment	109.11	O	X	Positive	1
				$C_{27}H_{45}$	[M+H-H_2O]+	369.35	O	X	Positive	1
				$C_{27}H_{43}O$	[M]-	383.33	O	X	Negative	3
				$C_{27}H_{46}O$	[M-H]-	385.37	O	X	Negative	1
	Glycerophospholipids (GP)	Glycerophosphocholines	GPCho	C_3H_9N	Fragment	59.02	X	O	Negative	1
	Others	-	[Na_2Cl]+	Na_2Cl	[M]+	80.95	O	X	Positive	6
		-	Hydroxyltropylium ion	C_7H_6OH	[M]+	107.09	O	X	Positive	6
		-	$C_{10}H_{10}N$	$C_{10}H_{10}N$	[M]+	144.1	O	X	Positive	4
		-	Tryptophan	$C_{10}H_{11}N_2$	[M]+	159.14	O	X	Positive	4

References

1. Passarelli, M. K. & Winograd, N. Lipid imaging with time-of-flight secondary ion mass spectrometry (ToF-SIMS). *Biochim. Biophys. Acta - Mol. Cell Biol. Lipids* **1811,** 976–990 (2011).

2. Tian, H. *et al.* Spatiotemporal lipid profiling during early embryo development of Xenopus laevis using dynamic Time-of-Flight Secondary Ion Mass Spectrometry (ToF-SIMS) Imaging. *J. Lipid Res.* **44,** 1–34 (2014).

3. Carlred, L. *et al.* Simultaneous imaging of amyloid-β and lipids in brain tissue using antibody-coupled liposomes and time-of-flight secondary ion mass spectrometry. *J. Am. Chem. Soc.* **136,** 9973–9981 (2014).

4. Gostek, J. A. *et al.* Differentiation between single bladder cancer cells using PCA of ToF-SIMS mass spectra. *Anal. Chem.* **87,** 3195–201 (2015).

5. Hanrieder, J. *et al.* High resolution metabolite imaging in the hippocampus following neonatal exposure to the environmental toxin BMAA using ToF-SIMS. *ACS Chem. Neurosci.* **5,** 568–575 (2014).

6. Hanrieder, J., Malmberg, P., Lindberg, O. R., Fletcher, J. S. & Ewing, a G. Time-of-flight secondary ion mass spectrometry based molecular histology of human spinal cord tissue and motor neurons. *Anal. Chem.* **85,** 8741–8748 (2013).
